# Asynchronous Bilateral Pneumothorax in a Patient With Birt-Hogg-Dubé Syndrome: A Case Report

**DOI:** 10.7759/cureus.96356

**Published:** 2025-11-08

**Authors:** Anna Eleftheriou, George Benakis, Anna Orfanidou, Sofia Theotokoglou, Dimitrios Lioumpas

**Affiliations:** 1 Department of Thoracic Surgery, Agios Panteleimon General Hospital Of Nikaia, Athens, GRC; 2 Department of Pathology, National and Kapodistrian University of Athens School of Medicine, Attikon General University Hospital, Athens, GRC; 3 Department of Dermatology and Venereology, National and Kapodistrian University of Athens School of Medicine, Attikon General University Hospital, Athens, GRC

**Keywords:** birt-hogg-dubé syndrome (bhds), diagnostic genetic testing, flcn gene, pulmonary cysts, spontaneous pneumothorax

## Abstract

Birt-Hogg-Dubé (BHD) syndrome is a rare autosomal dominant genetic disorder caused by mutations in the Folliculin (FLCN) gene and characterized by cutaneous fibrofolliculomas, multiple pulmonary cysts, spontaneous pneumothorax, and renal tumours. Pulmonary involvement is frequent and may represent the earliest manifestation of BHD. Due to its variable presentation, diagnosis is often delayed or missed.

We report the case of a 38-year-old woman with a known history of multiple sclerosis who presented with recurrent, asynchronous, bilateral, spontaneous pneumothoraces over a 15-month period, including during pregnancy. Imaging revealed bilateral apical lung air-filled cysts. Management included conservative treatment, chest tube insertion, and ultimately bilateral video-assisted thoracoscopic surgery (VATS) bullectomy and apical pleurectomy. During a multidisciplinary evaluation during pregnancy, numerous white to skin-coloured waxy papules were noticed, prompting a dermatologic consultation. The diagnosis of BHD syndrome was confirmed via skin biopsy. Family screening identified the same condition in her mother. Renal imaging showed no abnormalities, but long-term surveillance was advised.

This case highlights the potential for misdiagnosis or delayed diagnosis of BHD syndrome in patients presenting with spontaneous pneumothorax. A detailed family history, early recognition, and multidisciplinary evaluation are essential for early and appropriate management.

## Introduction

Birt-Hogg-Dubé (BHD) syndrome is a rare autosomal dominant genetic disorder caused by mutations in the Folliculin gene (FLCN), located on chromosome 17 [1]. The folliculin protein encoded by this gene participates in several signalling pathways involved in the regulation of cellular metabolism [2]. Clinically, BHD presents with a broad spectrum of manifestations, most commonly affecting the skin, lungs, and kidneys. Cutaneous fibrofolliculomas, multiple pulmonary cysts, spontaneous pneumothorax, and renal tumours are all possible manifestations of this disorder [3].

Pulmonary involvement is frequent and sometimes represents the earliest manifestation of BHD. Lung cysts in patients with BHD may involve up to 30% of the lung parenchyma, are often numerous, and may even be bilateral [1]. The prevalence of spontaneous pneumothorax in this population is estimated at 61%, with higher rates reported in the Asian population [4]. The median age of the first occurrence of spontaneous pneumothorax is approximately 38 years [1].

The present report demonstrates a case of asynchronous bilateral spontaneous pneumothorax in a patient with genetically confirmed BHD syndrome, highlighting the diagnostic challenges.

## Case presentation

**Figure 1:**
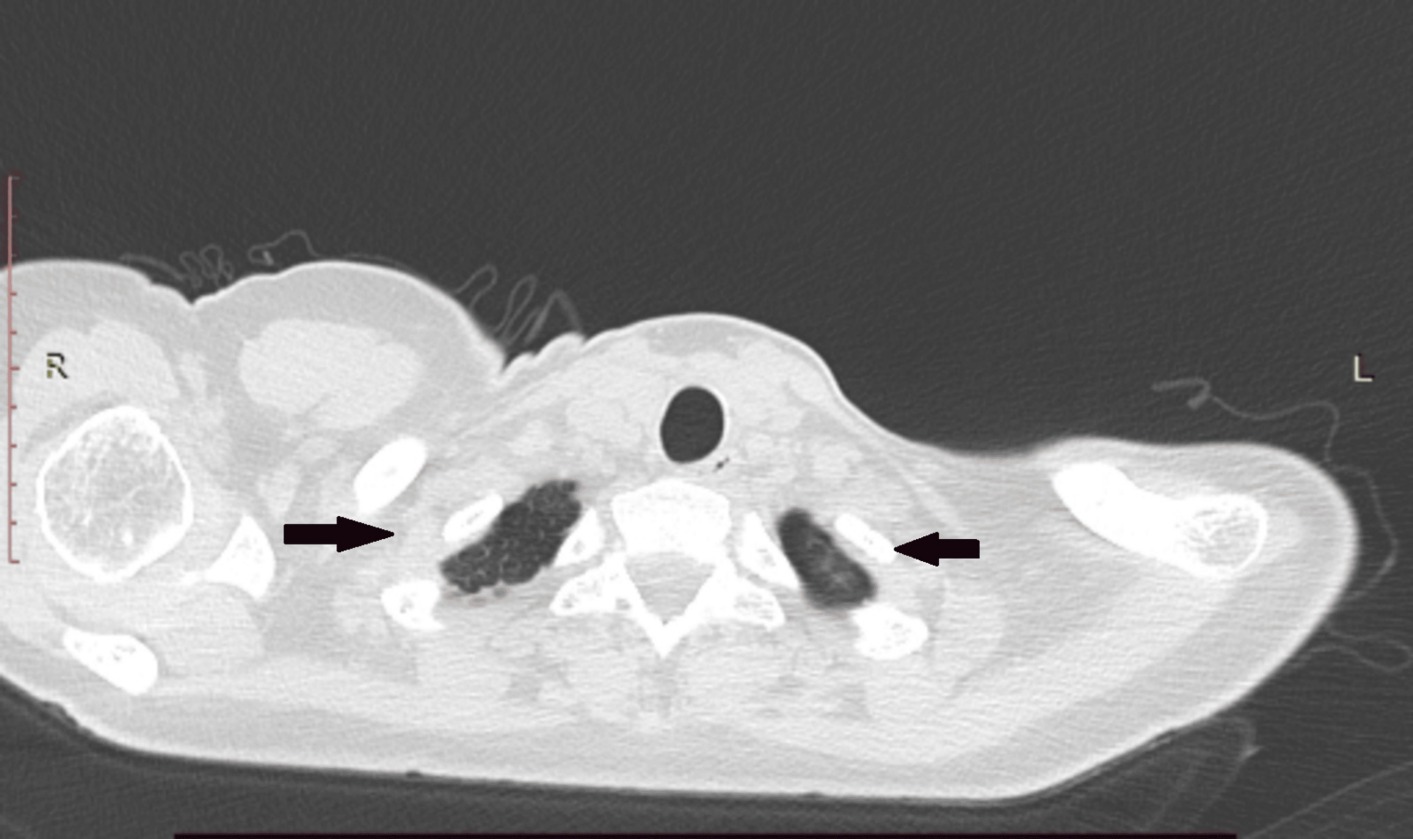
CT revealing multiple bilateral apical lung cysts after the first episode of spontaneous pneumothorax

A 38-year-old woman with a known history of multiple sclerosis (MS), untreated for the preceding two years, presented to the emergency department with acute chest pain. A chest radiograph revealed a left-sided spontaneous pneumothorax, considered her first episode, and a chest tube was placed. A computed tomography (CT) revealed multiple bilateral apical lung cysts (Figure 1).

The patient was discharged following full re-expansion of the affected lung. 

**Figure 2:**
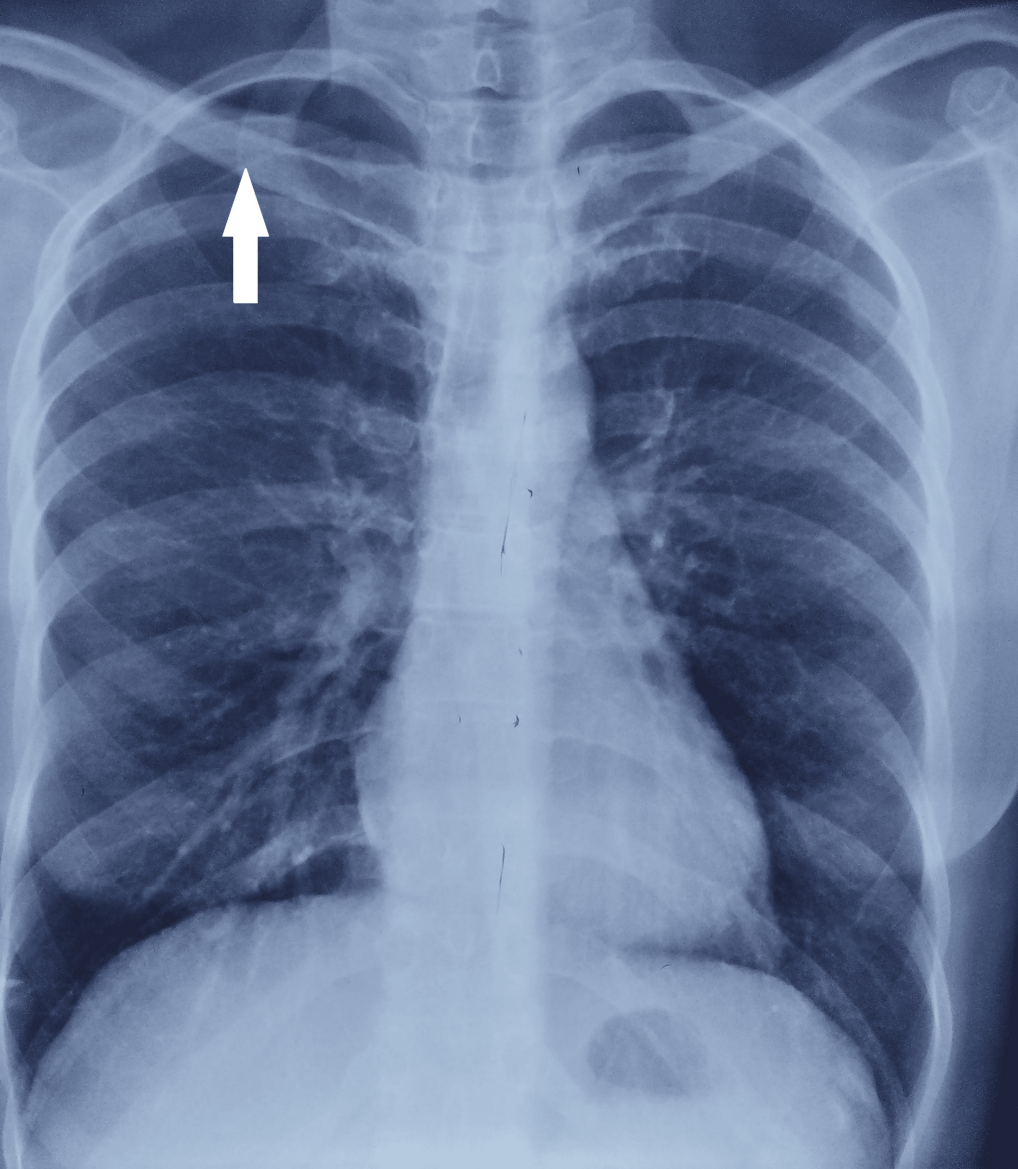
Radiograph showing right-sided pneumothorax

Three months later, the patient experienced right upper quadrant abdominal pain. A chest radiograph revealed a small apical right-sided pneumothorax (Figure 2).

As the pneumothorax was estimated to be less than 5% in size, no immediate intervention was deemed necessary. 

A month later, while eight weeks pregnant, the patient presented with a new onset of chest pain. Imaging showed a small, stable right-sided spontaneous pneumothorax, unchanged from the previous radiograph. She was managed conservatively with high concentration oxygen therapy. She improved clinically and was discharged after two days.

Five days post-discharge, the patient re-presented in the emergency department with symptoms of dry cough, dyspnoea and chest pain. Chest radiograph revealed an extensive left pneumothorax and a 28F chest tube was inserted. Given her history of MS, following clinical improvement, the patient was prompted to our hospital for multidisciplinary management.

During her hospitalization, the patient exhibited persistent air leak for over five days, prompting surgical intervention. Video assisted thoracoscopic surgery (VATS) bullectomy and apical pleurectomy was performed. Recovery was uneventful and the patient was discharged three days post-operatively.

**Figure 3:**
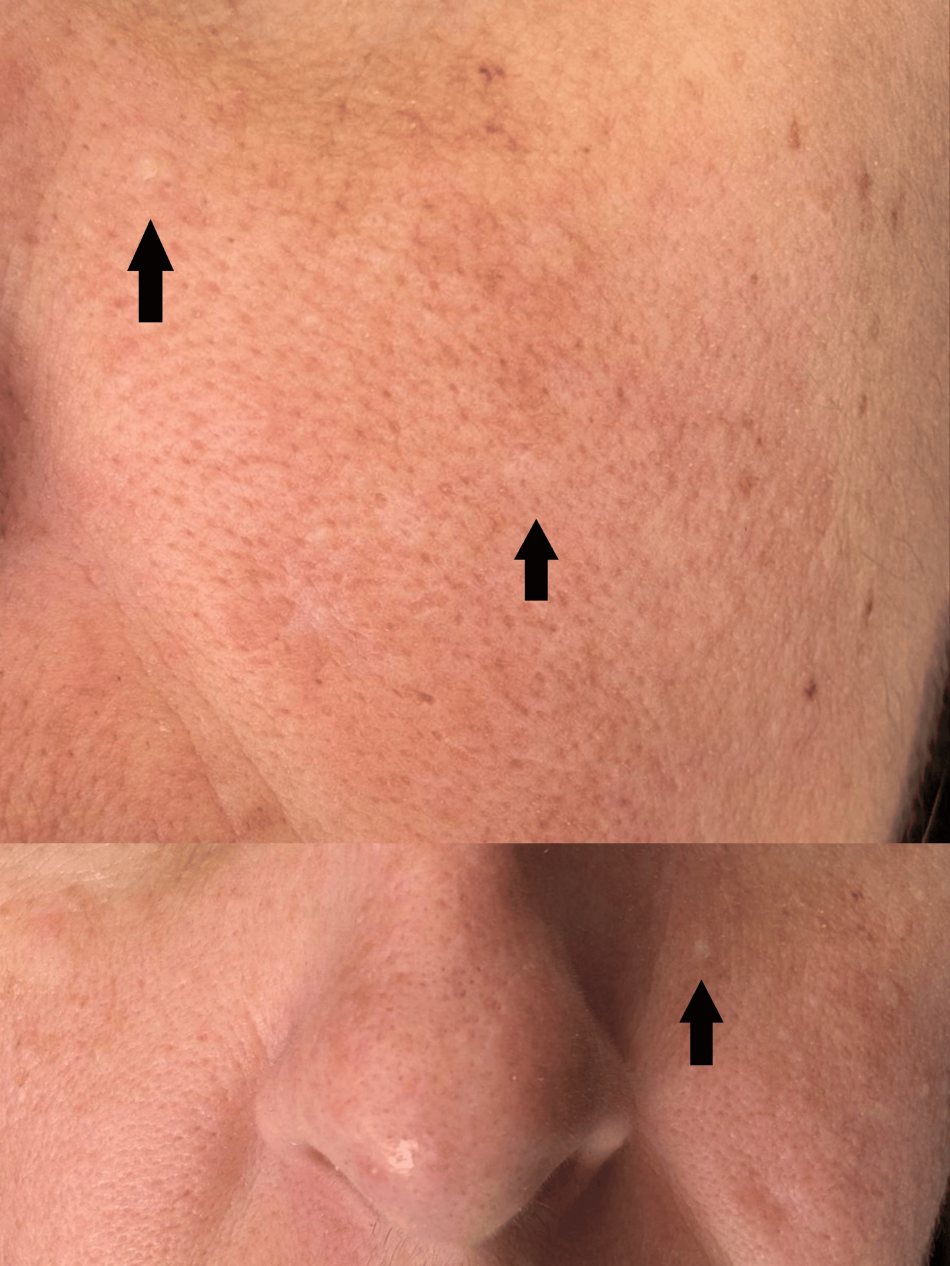
Numerous white to skin-coloured, waxy papules on the cheeks and nose

Due to the patient’s history of recurrent spontaneous pneumothoraces, it was recommended that an elective cesarean section be performed in a hospital facility with both thoracic surgery and obstetrics department. During hospitalization, a pulmonology consultation revealed multiple facial papules. Dermatologic evaluation showed numerous white to skin-coloured, waxy papules on the cheeks and nose (Figure 3), present since the age of 35.

**Figure 4:**
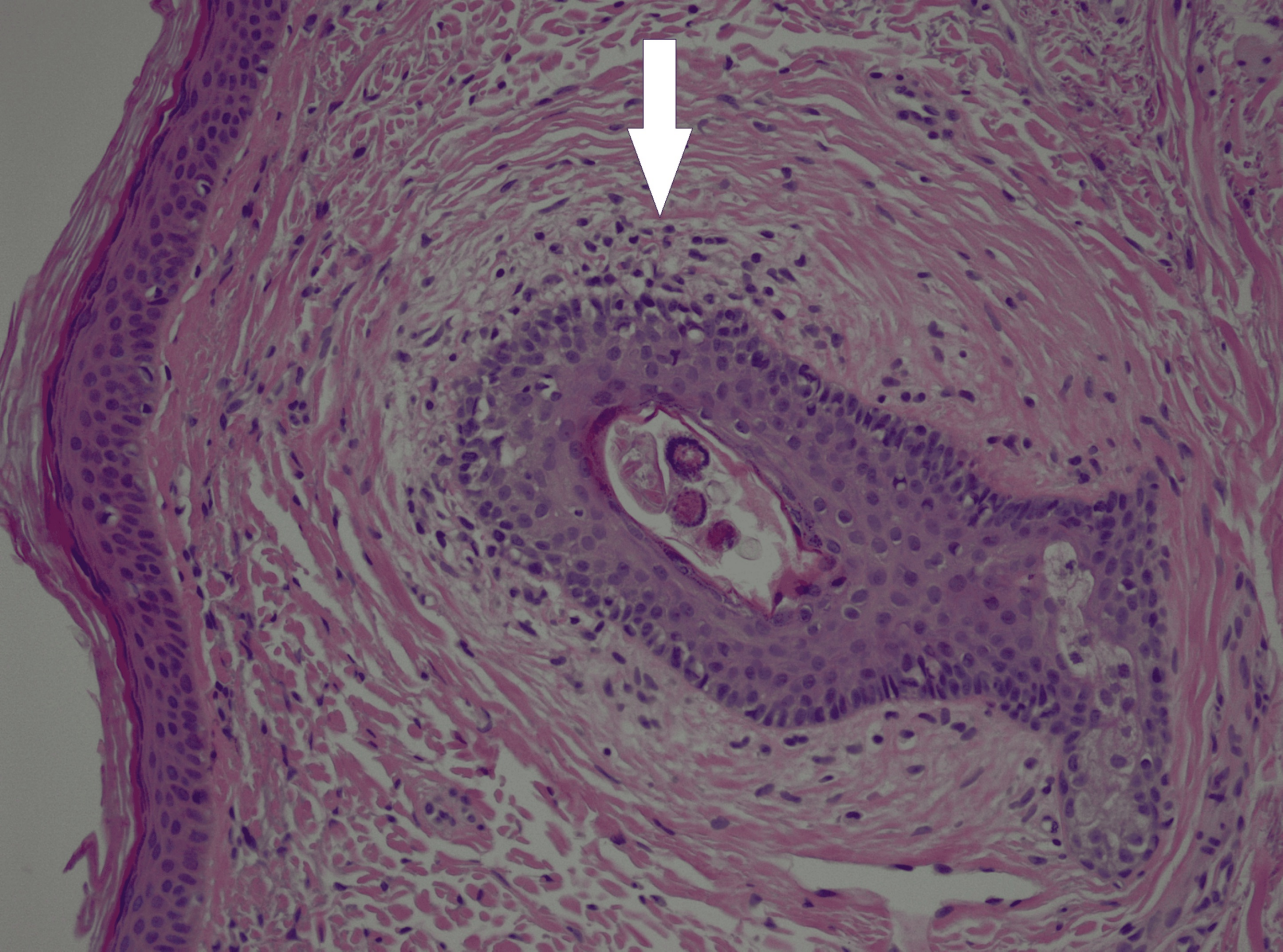
Histological confirmation of BHD syndrome A dense connective tissue sheath surrounds a normal hair follicle (onion skin effect); BHD: Birt-Hogg-Dubé.

A skin biopsy confirmed the diagnosis of BHD (Figure 4).

Renal ultrasound was negative for cysts or tumors.

**Figure 5:**
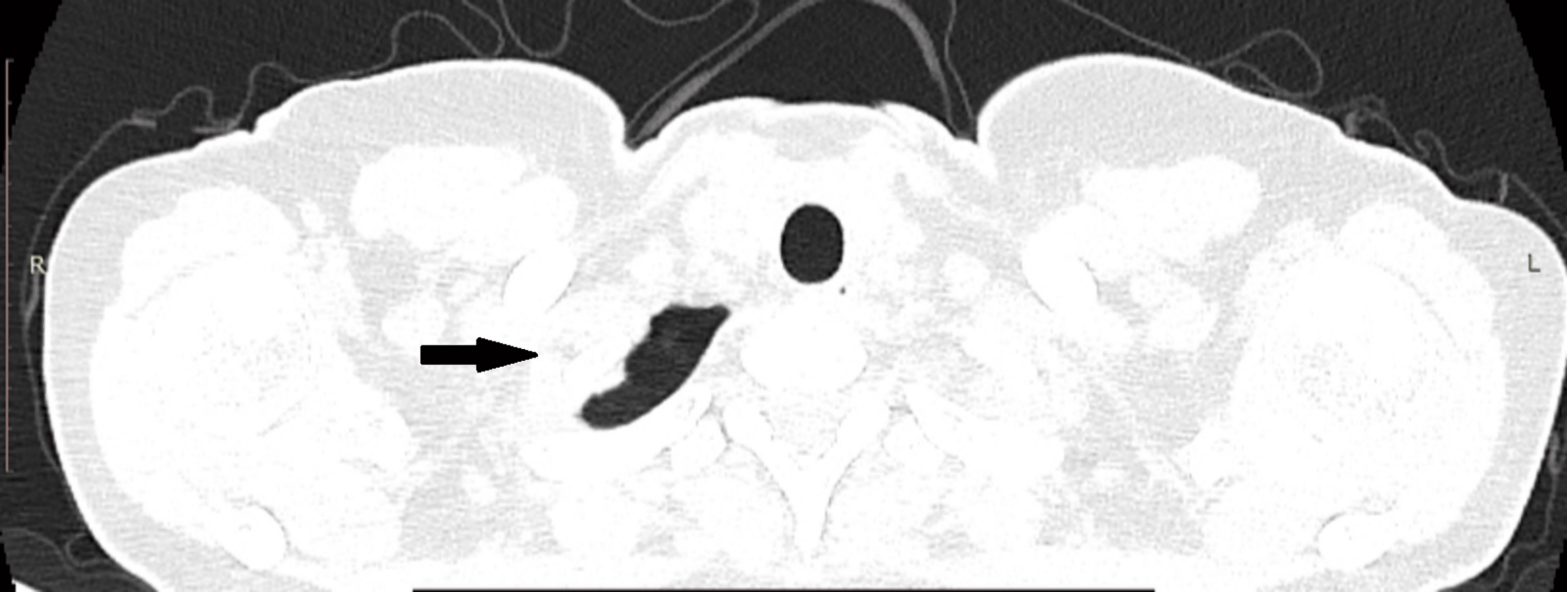
CT revealing a small right pneumothorax

Approximately one year after her last episode, a routine surveillance chest CT revealed a small right pneumothorax (Figure 5).

**Figure 6:**
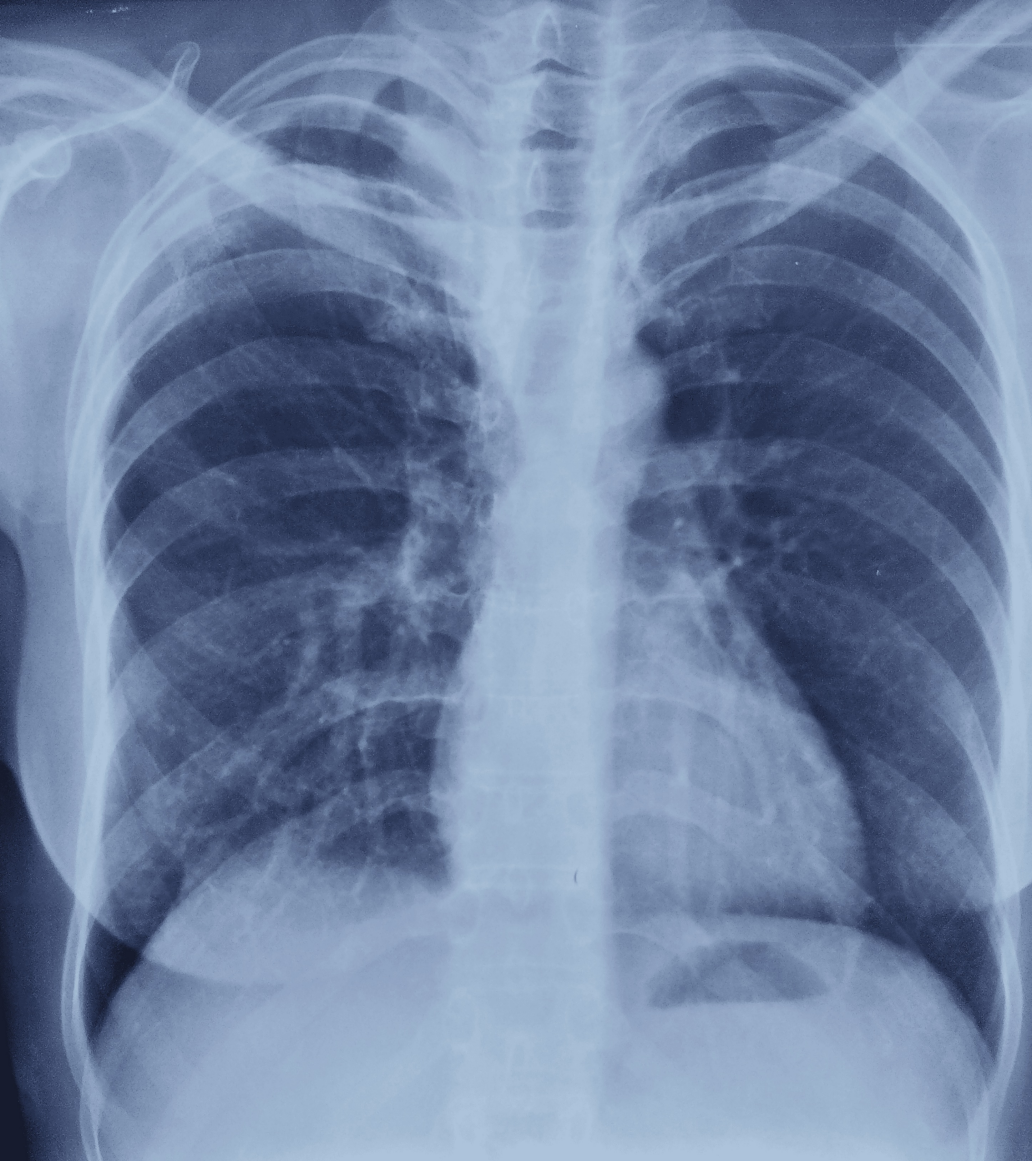
Follow-up chest radiography with satisfactory lung re-expansion

Because the patient was asymptomatic, no intervention was undertaken and a recommendation for repeat imaging was suggested. On follow-up, no improvement was noted and thus surgical intervention was deemed necessary. A scheduled VATS procedure for right persistent pneumothorax was performed and the patient was discharged three days post-operatively. At one-month follow-up, the chest radiograph demonstrated satisfactory lung re-expansion (Figure 6). 

Notably, one month later, her mother presented with a right-sided pneumothorax. Further evaluation confirmed BHD syndrome, establishing a positive family history.

## Discussion

BHD syndrome is frequently misdiagnosed as primary spontaneous pneumothorax or emphysema, due to its rarity and limited clinical awareness [1]. In our case, multiple episodes occurred before the diagnosis was made. The patient’s family history must always be taken into consideration, and physicians should include BHD syndrome in the initial differential diagnosis, particularly when skin lesions or renal cysts are present. Absence of such findings should not exclude the diagnosis of BHD, especially in patients with recurrent or bilateral pneumothorax and a positive family history [1], as spontaneous pneumothorax may in some cases be the only clinical manifestation [2].

In BHD syndrome, high-resolution computed tomography (HRCT) often reveals multiple thin-walled air-filled lung cysts, located primarily in the basal and subpleural areas of the lung. The lung parenchyma surrounding the cystic formations is typically normal, and thus pulmonary function often remains preserved. These characteristics may help distinguish BHD from other cystic lung diseases such as lymphangioleiomyomatosis, Langerhans cell histiocytosis or amyloidosis. Definitive diagnosis requires genetic confirmation or characteristic histopathology from skin biopsy, as it guides renal surveillance and family screening [1].

Renal cysts are observed in 19.2-45% of patients, while neoplastic transformation may also develop after presenting with bilateral multifocal tumours of varying sizes [5]. Among these, renal cell carcinoma (RCC) is considered the most serious manifestation, with cases reported even in patients younger than 25 years old, as the risk of developing tumours is estimated to be seven times higher than that of the general population [6,7]. Histologically, these tumours are most often hybrid oncocytic neoplasms which combine features of chromophobe RCC and renal oncocytoma. Chromophobe RCC, clear cell RCC, and renal oncocytoma are also reported [6]. Current recommendations suggest surveillance imaging every 36 to 48 months in the absence of findings. A renal mass ≥3 cm is generally considered an indication for surgical resection [1].

## Conclusions

This case highlights the risk of misdiagnosis or delayed recognition of BHD syndrome in patients presenting with spontaneous pneumothorax. As the full clinical spectrum may not be evident at the time of initial evaluation, maintaining a high index of suspicion is crucial, particularly in individuals with recurrent pneumothorax or a relevant family history. 

Early diagnosis of BHD syndrome is essential not only for the prevention of further pulmonary complications but also for the management of the associated risk for renal tumours. Early recognition allows for appropriate counselling, initiation of long-term renal surveillance, and ultimately improved outcomes for patients. A multidisciplinary approach is vital to ensure coordinated care for these patients.
